# A Study on the Awareness and Perceptions Regarding Monosodium Glutamate and its Potential Health Effects Amongst the Urban Population of Saudi Arabia

**DOI:** 10.7759/cureus.51094

**Published:** 2023-12-25

**Authors:** Muazzam M Sheriff, Hanin H Abusabah, Heba B Sindi, Alanoud O Alaidrous, Abdelmalik H Moemen, Shahad F Alshalawi, Bayan F Alshalawi, Nooraa Y Aljaoser, Lama K Alghamdi, Razan M Badri, Layan A Gadi, Safaa D Alotaibi, Ghada H Alharbi, Nawaf M Aljadani

**Affiliations:** 1 Microbiology and Immunology, Ibn Sina National College for Medical Studies, Jeddah, SAU; 2 Medicine, Ibn Sina National College for Medical Studies, Jeddah, SAU; 3 Medicine, University of Jeddah, Jeddah, SAU; 4 Clinical Dietitian Resident, King Fahad General Hospital, Jeddah, SAU; 5 Clinical Dietitian, National Guard Hospital, Jeddah, SAU; 6 Pediatric Service Resident, King Abdullah Medical Complex, Jeddah, SAU

**Keywords:** public health and safety, cardio vascular disease, fast food consumption, p-value, saudi arabia, urban population, cross section study, intricacies, chinese salt, monosodium glutamate (msg)

## Abstract

Introduction

Monosodium glutamate (MSG), a common global food additive in processed foods, influences flavors and textures due to its chemical complexity and nutritional intricacy. Despite an annual production of 1.9 million tons and historical safety concerns, the multifaceted impact on health, ranging from metabolic disorders to neurological and cardiovascular implications, necessitates ongoing research for informed consumption and balanced dietary practices.

Materials and methods

This cross-sectional study investigates MSG-associated intricacies among Saudi Arabia's urban population. The research included questionnaire development, translation, and cultural adaptation, and was validated by nutrition experts. A sample size of 420 was calculated for a 95% confidence level. Data collection occurred from September 13 to October 31, 2023, and ethical considerations were ensured. Statistical analysis, including chi-square tests, regression analysis, and SPSS, explored intricacy relationships.

Results

The MSG intricacy study in Saudi Arabia's urban population, involving 420 respondents, showed statistically significant correlations (P < 0.05) in demographics. The key findings indicate an awareness of the impact of MSG on health, its associations with various conditions, and strong support for its exclusion from foods. Region, gender, age, and social status correlations highlighted diverse perspectives. The Western province showed the highest response rate at 42.61%, prompting regional awareness questions. Gender dynamics showed that 90.47% of the respondents were females, emphasizing potential gender-specific concerns. Concentration among ages 20-30 (61.9%) underscored generational factors. While commendable baseline awareness was noted, 73.09% of the participants believing MSG is harmful prompts further investigation. Emotional responses, including happiness (25.95%) and frustration (18.33%), highlight the complexity of the individuals' experiences, emphasizing the need for tailored communication strategies.

Conclusion

The MSG intricacy study in Saudi Arabia's urban population reveals insights into knowledge, attitudes, and behaviors, emphasizing the need for nuanced interventions considering regional and emotional differences. The findings underscore health concerns, supporting regulations, and knowledge impact on behavior. This survey serves as a valuable tool for informed public discourse and decision-making in the unique socio-cultural context of urban Saudi Arabia.

## Introduction

Monosodium glutamate (MSG), also referred to as E621 or Chinese salt, is a pervasive and globally prevalent food additive seamlessly incorporated into processed foods, displaying both perplexity and bustiness in its chemical composition [1]. Chemically, MSG is identified as the sodium salt of glutamic acid, denoted as Sodium 2-aminopentanedioate according to the International Union of Pure and Applied Chemistry (IUPAC) nomenclature. Upon dissolution in water, MSG transforms into glutamic acid and sodium ions, characterized by a structural composition featuring an alpha carbon atom bonded to an amino group (-NH_2_) and a carboxylic group (-COOH). Its physical manifestation is a white crystalline powder, resembling salt or sugar [2]. Classified as a non-essential amino acid, MSG occurs naturally in a myriad of foods, exhibiting its versatility in meats, seaweed, anchovies, molluscs, tomatoes, cheeses, vegetables, and shellfish, as well as human and cow milk, apples, almonds, eggs, onions, carrots, potatoes, walnuts, and garlic. The incorporation of MSG in processed foods such as meats, crackers, frozen meals, soups, salad dressings, baby formula, canned tuna, fast food, frozen dinners, and potato chips adds to its presence in the modern diet [3]. Despite historical safety concerns, MSG remains a regular component, contributing to a perplexing interplay of flavors and textures [4]. Annually, a staggering 1.9 million tons of MSG are produced globally through a fermentation process, primarily utilizing *Corynebacterium glutamicum *or closely related species [5]. Bera and co-workers emphasize salt (NaCl) and MSG as primary active constituents in flavour enhancers, reflecting the intricate and dynamic nature of their composition [6]. The metabolic journey of glutamate within the gastrointestinal tract adds a layer of complexity to its perplexing role. Moreover, the inclusion of additional minerals in MSG salts contributes to a metallic and umami flavour, with sodium glutamate exhibiting superior solubility and crystallization rates, accentuating the intricate sensory experience associated with its consumption [7]. In terms of production methods and metabolism, genetically modified bacteria have been employed since 1957 in the United States to produce MSG from sources such as sugarcane molasses and carbohydrates like corn, showcasing the technological business in its manufacturing [8]. The metabolic processing of MSG within the human body occurs in the small intestine and involves specialized transporters facilitating its journey alongside other amino acids [9]. Once absorbed into the bloodstream, glutamate undergoes intricate metabolic processing and contributes to energy production, amino acid conversion into glucose through gluconeogenesis, and the synthesis of essential molecules [10].

The consumption of MSG has been intricately associated with a range of health issues, underscoring both perplexity and role of its impact on metabolic disorders [11]. Prolonged MSG intake has been linked to various metabolic diseases, including diabetes, dyslipidemia, and obesity, along with reported associations with cardiovascular diseases, sleep and respiratory disorders, neuroendocrine defects, depression, and anxiety [12]. The adverse effects extend to genotoxicity, hepatotoxicity, renal toxicity, and reproductive toxicity, indicating an intricate web of health implications [13]. MSG's neurotoxic properties have been implicated in the development of pathological disorders such as Parkinson's disease, depression, stroke, brain injury, anxiety, addiction, Alzheimer's disease, and epilepsy, showcasing the diverse and complex nature of its impact on neurological health [14]. Research into MSG's role in diabetes mellitus reveals disruptions in glucose absorption, resulting in hyperglycemia within a defined timeframe, potentially linked to decreased pancreatic beta-cell mass, increased hemorrhages, and fibrosis [15]. The association between MSG and hypertension, a significant contributor to cardiovascular diseases, further adds to its role in health implications. Studies in regions with high hypertension prevalence highlight the widespread use of MSG in local cuisine, correlating with increased fat and sodium intake and higher blood pressure, particularly in women and non-smokers [16]. Even individuals without hypertension demonstrate adverse effects, emphasizing the unexpected and diverse impact of MSG on health. A study published in the journal "Cephalalgia," uncovered a momentary rise in blood pressure in people who consumed elevated quantities of MSG, such as sugar-free soda [17]. This finding provides valuable information about the immediate effects of MSG consumption on blood pressure. Daily intake of high MSG doses may lead to fatigue, underscoring the role of its impact on daily well-being [18]. Despite being a widely used food additive, MSG, particularly in excessive amounts, continues to be associated with a spectrum of health implications, necessitating ongoing research to unravel the intricate metabolic pathways and potential risks associated with its consumption [19]. This highlights the importance of balanced dietary practices and informed consumption in navigating the complexities of the effects of MSG on health [20].

The study on the intricacies of monosodium glutamate (MSG) within the urban population of Saudi Arabia is driven by a compelling need to gain a comprehensive understanding of individuals' awareness, perceptions, and behaviors concerning this pervasive food additive. The global prevalence of MSG as a ubiquitous component in processed foods necessitates a closer examination, especially considering ongoing concerns about its potential health implications despite its widespread use. MSG's chemical complexity and nutritional intricacy, influencing both taste and nutritional profiles, highlight the necessity of understanding its intricate nature for promoting informed consumption and fostering balanced dietary practices [21]. With MSG being associated with a spectrum of health issues, from metabolic disorders to neurological and cardiovascular implications, the study aims to explore its multifaceted impact on health, addressing concerns that extend to metabolic diseases and cardiovascular and neurological disorders [17]. The lack of comprehensive studies focusing on the urban population of Saudi Arabia emphasizes the significance of this research, intending to provide valuable insights into the awareness and perceptions of individuals within this specific socio-cultural context. The study recognized and explored regional and demographic variances, acknowledging the diversity of the urban Saudi population and tailoring interventions accordingly. Given the historical safety concerns surrounding MSG, there is a pressing need for informed consumer choices. The study contributed to public health initiatives by providing intricacies insights into factors that influence MSG awareness and behaviors. Additionally, exploring cultural and emotional intricacies associated with food additives contributed to a more holistic understanding of MSG's role in the urban Saudi Arabian context. The study's findings served as a valuable tool for informed public discourse and decision-making, shedding light on MSG intricacies and contributing to evidence-based policies and educational initiatives for promoting informed and balanced dietary practices in this culturally rich environment. Although MSG remains a common food additive, its consumption, especially in excessive quantities, has been linked to a range of health consequences [21]. Current research endeavors persist in delving into the complex metabolic pathways and potential health risks connected to MSG, underscoring the significance of maintaining balanced dietary practices and making informed choices in consumption. 

Aims and objectives of the research

This study was designed with clear objectives to comprehensively investigate the intricacies surrounding MSG among the urban population of Saudi Arabia. The primary aim was to assess the diverse health implications associated with MSG consumption, delving into potential links with metabolic disorders, cardiovascular issues, and neurological conditions. Additionally, the research seeks to gauge the level of awareness and perceptions among individuals in urban Saudi Arabia regarding MSG, exploring their understanding of its impact on health and associations with various conditions. Examining behaviors related to MSG consumption, including dietary habits, was another crucial objective. Demographic parameters, such as age, gender, social status, and regional variations, were analyzed to understand how these factors shaped perspectives on MSG. The study also aimed to contribute valuable insights to public health initiatives by informing and educating the population, thereby facilitating informed decision-making and potentially influencing evidence-based policies. Furthermore, the exploration of cultural and emotional nuances associated with MSG consumption provided a holistic understanding, emphasizing the need for tailored communication strategies in this unique socio-cultural context. Overall, these objectives collectively aimed to fill research gaps and offer a comprehensive understanding of MSG intricacies in the urban landscape of Saudi Arabia.

## Materials and methods

Research design

This study employs a cross-sectional research design to investigate the intricacies associated with the consumption of MSG among the urban population of Saudi Arabia. The research methodology encompasses the creation and validation of a questionnaire, data collection, and subsequent analysis [22].

Questionnaire development

A comprehensive questionnaire was developed to assess the population's knowledge of MSG intricacies. The questionnaire included sections covering demographic details, intricacy-related variables, psychosocial aspects, and validated assessment scales [23].

Translation and cultural adaptation

To ensure cultural relevance and linguistic accuracy, a meticulous translation and adaptation process was undertaken for the questionnaire. Bilingual experts translated the English version into Arabic, followed by a separate back-translation by a different set of bilingual experts [24]. Consensus resolution addressed any discrepancies, maintaining the original intent while aligning with the local cultural context [25].

Questionnaire validation

The translated questionnaire underwent validation to ensure reliability and validity. Content validation involved a panel of nutrition experts. A pilot study on a small sample assessed the clarity and comprehensibility of questionnaire items, with participant feedback used to refine it.

Sample size

The study's sample size was determined using the Rao soft online calculator, aiming for a 95% confidence level with a margin of error within ±5%. The calculated sample size required was 420, based on inclusion criteria set by a specialist nutrition health care professional [26].

Data collection

A cross-sectional study was implemented through an online survey utilizing a Google Form (in English), employing a non-probability convenience sampling technique. The survey outreach was facilitated through various social media platforms, namely Twitter, Telegram, and WhatsApp. Distribution involved sending the survey to the targeted population via groups and direct messages, ensuring widespread access for the participation. Informed consent was obtained, emphasizing confidentiality and voluntary participation. Sanctioned by the Institutional Human Ethics Committee, the study occurred from September 13, 2023, to October 31, 2023, with 420 participating volunteers. The primary objective was to assess the proportion of all ages.

Data analysis

Thorough statistical analysis was performed on the collected data. Descriptive statistics presented demographic and intricacy-related variables. Validated assessment scales were analyzed to quantify intricacy prevalence and severity [27]. Inferential statistics, including chi-square tests and regression analysis, explored relationships between intricacy and various factors. The statistical analysis was executed using the International Business Machines Statistical Package for the Social Sciences (IBM SPSS) Statistics 23 for Windows [25]. SPSS and Microsoft Excel were utilized for data analysis, maintaining a statistical power of 80% at a cutoff value of 0.05 [28]. The dual utilization of SPSS (Statistical Package for the Social Sciences) and Microsoft Excel software platforms was selected to harness the strengths of both tools, allowing for a comprehensive analysis of the dataset [27]. SPSS, renowned for its robust statistical capabilities, provided advanced analytical features, while Microsoft Excel offered a user-friendly interface for certain aspects of data manipulation and visualization [26].

Ethical considerations

This study strictly adhered to ethical guidelines, prioritizing participant anonymity, privacy, and informed consent. The study titled "A Study on the Intricacies of Monosodium Glutamate Amongst the Urban Population of Saudi Arabia" was approved by the Ibn Sina National College (ISNC) Institutional Research Review Board (IRRB) under ethical approval IRRB-01-03092023, with protocol identification number 001MP10082023

## Results

The participation of 420 respondents significantly contributed to the comprehensiveness and robustness of our dataset. The systematic acquisition of data served as a foundational element in our research methodology, guaranteeing a rigorous approach to managing the information obtained from the survey participants. The resulting dataset, a product of careful curation, was systematically presented in Table 1.

**Table 1:** Response Rate

Survey Questionnaires	Response Rate Absolute Number 420 participants	Response Rate Percentage (%)
Province		
Northern	43	10.2
Eastern	37	8.80
Western	179	42.61
Southern	89	21.19
Central	72	17.10
Gender		
Female	380	90.47
Male	40	9.53
Age		
Less than 20 year	30	7.1
20-30 year	260	61.9
30-50 year	110	26.19
Over 50 years old	20	4.76
Social status		
Single	180	42.85
Married	230	54.76
Divorced	10	2.38
Do you know what is MSG?		
Yes	309	73.57
No	90	21.42
May Be	21	5
Did you know that MSG used as a food flavour enhancer is harmful to health?		
Yes	307	73.09
No	92	21.90
May Be	21	5
Do you know that eating foods containing MSG may lead to high blood pressure?		
Yes	129	30.71
No	201	47.85
May Be	90	21.42
Do you know that consuming foods with MSG may be linked to the development of diabetes?		
Yes	19	4.52
No	351	83.57
May Be	50	11.90
Have you noticed that foods containing MSG might leads to more activity than normal?		
Yes	90	21.42
No	253	60.23
May Be	77	18.33
Have you noticed that foods that contain MSG can cause headaches?		
Yes	153	36.42
No	220	52.38
May Be	47	11.19
Do you have an idea that the consumption of MSG can lead to salt and water retention in the body?		
Yes	227	54.04
No	114	27.10
May Be	79	18.80
Do you have any idea that the consumption of MSG is associated with weight gain?		
Yes	163	38.80
No	159	37.85
May Be	94	22.38
Have you noticed that you have an addiction to fast food and energy drinks?		
Yes	221	52.61
No	148	35.23
May Be	51	12.1
Do you support the idea of excluding MSG from foods?		
Yes	359	85.47
No	35	8.33
May Be	26	6.19
Does your knowledge of the harmful effects of MSG help you to reduce its use?		
Yes	363	86.42
No	35	8.33
May Be	22	5.2
Does eating foods containing MSG make you happy?		
Yes	109	25.95
No	129	30.71
May Be	182	43.33
Does eating foods that contain MSG make you feel frustrated?		
Yes	77	18.33
No	159	37.85
May Be	184	43.8

The outcomes of our analysis were presented in percentage format to enhance clarity. The Pearson Chi-Square tests, a widely accepted statistical method, were employed to explore correlations between variables present in our dataset [24]. Key demographics like province, gender, age, and social status, coupled with attitudes toward MSG, were studied. Outcomes in Table 1, featuring responses with absolute numbers and percentages, showed statistically significant correlations. The regression analysis, involving 420 participants, detailed response rates across demographics. In provinces, the Western region led with 42.61%. Gender dynamics showed that 90.47% of the respondents were females, and the 20-30 age group represented 61.9% of the population. The majority of the participants were married (54.76%). MSG awareness was high, with 73.57% aware of MSG and its harmful effects. A substantial number of participants acknowledged MSG's potential impact on high blood pressure (30.71%), diabetes (83.57%), increased activity (21.42%), and headaches (36.42%). Concerns about salt/water retention (54.04%) and weight gain (38.80%) were evident. Strong support for excluding MSG from foods was recorded at 85.47%.

Table 2 succinctly summarises significant correlations. The p-values indicated significant associations, specifically with respect to province (0.052), gender (0.022), age (0.042), and social status (0.043). Awareness of MSG and attitudes also showed significant associations. Future research could delve deeper into factors influencing attitudes and informing targeted interventions. Comparing this report to another study, commonalities include exploration of MSG perceptions, cross-sectional design, and emphasis on nuanced interventions [16]. Differences lie in scope, demographics, MSG awareness details, health perceptions, behavioral aspects, support for regulations, impact on knowledge, emotional responses, and limitations. The other study, specific to the urban population of Saudi Arabia, provides detailed context-specific insights into MSG perceptions, enriching understanding in the Saudi Arabian context [20].

**Table 2:** p-Value

Survey Questionnaires	p-value (P ≤ 0.05)
Province	0.052
Gender	0.022
Age	0.042
Social status	0.043
Do you know what is MSG ?	0.025
Did you know that MSG used as a food flavor enhancer is harmful to health?	0.027
Do you know that eating foods containing MSG may lead to high blood pressure?	0.041
Do you know that consuming foods with MSG may be linked to the development of diabetes?	0.046
Have you noticed that foods containing MSG might lead to more activity than normal?	0.036
Have you noticed that foods that contain MSG can cause headaches?	0.039
Do you have an idea that the consumption of MSG can lead to salt and water retention in the body?	0.047
Do you have any idea that the consumption of MSG is associated with weight gain?	0.044
Have you noticed that you have an addiction to fast food and energy drinks?	0.041
Do you support the idea of excluding MSG from foods?	0.024
Does your knowledge of the harmful effects of MSG help you to reduce its use?	0.029
Does eating foods containing MSG make you happy?	0.033
Does eating foods that contain MSG make you feel frustrated?	0.031

Table 2 provides a summary of the noteworthy findings arising from our statistical analysis. Each p-value corresponds to a specific variable, and the null hypothesis assumes that there is no significant association between the variable and the survey response. The examination of various factors involved the application of statistical tests tailored to each context. These include the use of the chi-square test for variables such as province (p = 0.052), gender (p = 0.022), age (p = 0.042), social status (p = 0.043), knowledge of MSG (p = 0.025), Knowledge of the harmful effects of MSG (p = 0.027), knowledge of the relationship between MSG and high blood pressure (p = 0.041), Knowledge of MSG and Diabetes (p = 0.046), MSG and Increased Activity (p = 0.036), MSG and Headaches (p = 0.039), MSG and salt/water retention (p = 0.047), MSG and weight gain (p = 0.044), addiction to fast food and energy drinks (p = 0.041), support for excluding MSG from foods (p = 0.024), knowledge of the impact of reducing MSG use (p = 0.029), MSG and happiness (p = 0.033), and MSG and frustration (p = 0.031). These statistical tests contribute to a comprehensive understanding of the respondents' characteristics, knowledge, and perceptions regarding monosodium glutamate (MSG) consumption.

This study, characterized by a robust sample size, meticulous data organization, and a rigorous dual-software analytical approach, stands as a significant contribution to the field. The findings presented in Table 2 offer a clear and concise representation of the correlations identified through our statistical analysis.

## Discussion

The outcomes of our survey offer a comprehensive understanding of participants' awareness and perceptions regarding MSG and its potential health effects. Notably, the demographic distribution discloses intriguing patterns, with the Western province taking a substantial lead at 42.61%, prompting a closer examination of regional patterns of awareness of the effects of MSG. Gender dynamics showed that 90.47% of the respondents were females, raising questions about potential gender-specific concerns or interests related to MSG. The concentration of respondents between the ages of 20-30 (61.9%) hints at a potentially heightened impact on younger individuals, emphasizing the importance of understanding generational factors that shape attitudes toward MSG within this demographic.

The study provides a commendable baseline awareness level, with 73.57% of participants familiar with what MSG is. However, the finding that 73.09% of the participants believe MSG, when used as a food flavor enhancer, is harmful calls for a deeper investigation into the sources influencing this perception. Unraveling the basis of this belief can provide crucial insights into the information channels shaping public opinion on food additives, and shed light on the factors contributing to the prevailing attitudes toward MSG.

Table 1 encapsulates response rates and key findings, revealing regional variations, gender dominance, and prevalent awareness of MSG's health effects. Noteworthy associations, such as province, gender, age, social status, and various aspects of MSG awareness and its effects, were observed.

Comparing this study with others, commonalities lie in the exploration of MSG perceptions, cross-sectional design, and nuanced interventions [16]. Differences are evident in the scope, demographics, details of MSG awareness, health perceptions, behavioral aspects, support for regulations, impact on knowledge, emotional responses, and limitations.

The p-values in Table 2 reflect the statistical significance of relationships between variables and responses. The dual-software approach, utilizing SPSS for advanced analytics and Excel for user-friendly manipulation, enhances the study's reliability and replicability.

The exploration of perceived health effects adds complexity to the narrative, revealing a growing awareness of potential cardiovascular implications, as evidenced by 30.71% associating MSG with high blood pressure. A study published in Cephalalgia revealed a temporary increase in blood pressure among individuals consuming high amounts of MSG, such as in sugar-free soda [17]. Chronic daily intake of high MSG doses may also lead to fatigue. Conversely, the smaller percentage associating MSG with diabetes (4.52%) highlights a gap in public understanding, emphasizing the need for targeted education on the broader health implications of food additives [29]. The revelation that 21.42% of participants feel more active after consuming MSG-containing foods introduces a novel dimension, prompting further research into the physiological effects of MSG and its impact on individuals. Prevalent concerns about headaches (36.42%), salt and water retention (54.04%), and weight gain (38.80%) form a complex network of perceived health risks associated with MSG consumption, indicating the multifaceted nature of public apprehensions. This suggests that health campaigns should address a spectrum of potential impacts to resonate with diverse audience concerns and provide comprehensive information to the public.

Behavioral aspects revealed in the survey unveil a noteworthy connection between MSG and addictive behaviors, with over half of the participants (52.61%) admitting to an addiction to fast food and energy drinks. This linkage necessitates a deeper exploration of the relationship between MSG and dietary choices, emphasizing the importance of considering broader lifestyle patterns in public health discussions [30]. The overwhelming support for excluding MSG from foods (85.47%) serves as a resounding call for regulatory measures or alternative options, indicating that the surveyed population perceives MSG as a significant health concern. Equally noteworthy is the substantial impact on knowledge, with 86.42% claiming that awareness of the harmful effects of MSG has influenced them to reduce its use. This finding underscores the potential efficacy of educational interventions in reshaping consumer behavior and fostering healthier dietary choices.

Emotional responses introduce a qualitative dimension to the survey, unveiling a spectrum of sentiments associated with MSG-containing foods. The fact that 25.95% feel happy, 18.33% feel frustrated, and 43.33% experience uncertainty or mixed feelings highlights the complexity of individuals' emotional experiences. This emotional variability underscores the importance of tailoring communication strategies that acknowledge and address diverse emotional responses. Understanding the emotional landscape surrounding MSG consumption is crucial for developing empathetic and relatable public health messaging that resonates with the diverse emotional states of the surveyed population.

Strengths

The study excels in achieving diverse representation within the urban population of Saudi Arabia, particularly focusing on the western province. This diversity enhances the generalizability of findings, providing a nuanced understanding of Monosodium Glutamate (MSG) perceptions across various urban contexts. The exploration of demographic factors, such as region, gender, and age, is a key strength, offering insights into potential regional and generational influences on MSG awareness. This demographic analysis allows for targeted interventions, amplifying the study's practical implications. A robust baseline awareness level, with over 73% familiar with MSG, establishes a solid foundation, while the inclusion of behavioral and emotional dimensions enriches the study's scope. Revelations about the connections between MSG and addictive behaviors, diverse emotional responses, and strong support for excluding MSG from foods underscore the study's societal relevance. Additionally, the study highlights a substantial impact on knowledge, with 86.42% claiming that awareness of MSG's harmful effects influenced them to reduce its use, emphasizing the potential effectiveness of educational interventions.

Weaknesses

Despite its strengths, the study faces notable weaknesses. Exclusive focus on the western province may introduce regional bias, limiting generalizability. A more extensive geographical representation is needed for a comprehensive national perspective. The significant gender imbalance (90.47% female) raises concerns about result skewing, necessitating a more balanced gender representation. While insightful, the emphasis on the 20-30 age group may limit broader applicability. Exploring a wider age range or distinct age groups could provide a nuanced understanding. The study identifies a significant percentage (73.09%) of the population believing that MSG is harmful but lacks an in-depth exploration of sources of these beliefs. Understanding the source of the beliefs (media, cultural influences, or personal experiences) is crucial. While the documentation of feelings after MSG consumption introduces a novel aspect, our study doesn't explore physiological effects extensively. Further investigation into reported experiences would enhance the depth of our study. While emotional responses are acknowledged, the study minimally explores qualitative data. Integrating qualitative methods, like interviews or focus groups, could provide richer insights into emotional dimensions associated with MSG consumption.

## Conclusions

The study on the awareness and perceptions regarding Monosodium Glutamate and its potential health effects within the urban population of Saudi Arabia provides insights into the knowledge, attitudes, and behaviors of participants. The findings underscore the need for targeted interventions that consider regional, demographic, and emotional nuances. The notable concerns about potential health effects, coupled with strong support for regulatory measures, and the impact of knowledge on behavior, underscore the significance of public health campaigns and educational initiatives tailored to the specific context of the urban population in Saudi Arabia. As we navigate the complex relationship between individuals and MSG, further research could delve into the intersection of cultural factors, media influence, and individual experiences to enhance our understanding of this multifaceted issue. Ultimately, the survey serves as a valuable tool for shaping informed public discourse and decision-making around the use of MSG in foods within the unique socio-cultural context of urban Saudi Arabia.
